# High Rates of Nonsusceptibility to Common Oral Antibiotics in *Streptococcus pneumoniae* Clinical Isolates From the United States (2019–2021)

**DOI:** 10.1093/ofid/ofae470

**Published:** 2024-08-22

**Authors:** Lalitagauri M Deshpande, Michael D Huband, Sarah Charbon, Mariana Castanheira, Rodrigo E Mendes

**Affiliations:** Element Iowa City (JMI Laboratories), North Liberty, Iowa, USA; Element Iowa City (JMI Laboratories), North Liberty, Iowa, USA; Element Iowa City (JMI Laboratories), North Liberty, Iowa, USA; Element Iowa City (JMI Laboratories), North Liberty, Iowa, USA; Element Iowa City (JMI Laboratories), North Liberty, Iowa, USA

**Keywords:** CABP, omadacycline, respiratory infections, SENTRY, *tet*(M)

## Abstract

*Streptococcus pneumoniae* isolates from the United States (n = 1038; 2019–2021) were susceptible to omadacycline (99.8%), levofloxacin (99.7%), and ceftriaxone (98.1%), whereas doxycycline (80.2%), oral penicillin (63.5%), cefpodoxime (76.8%), and azithromycin (54.4%) activity was limited. *Tet*(M) did not affect omadacycline activity but altered activity of older tetracyclines including doxycycline, suggesting omadacycline is an important option for treatment of community-acquired bacterial pneumonia.

*Streptococcus pneumoniae* is responsible for significant morbidity and mortality, especially among young children and older adults. Immunization policies with pneumococcal vaccines containing the most common serotypes have reduced incidence of infections and colonization with those serotypes, and reduced nonsusceptibility to multiple antimicrobial agents among pneumococcal isolates from adults with invasive and noninvasive pneumonia [1]. However, infections with serotypes not covered by vaccines, as well as breakthrough infections with vaccine serotypes, continue to evolve [2]. Delays in appropriate therapy and growing incidence of virulent/resistant strains can cause severe complications of bacterial pneumonia.

Community-acquired bacterial pneumonia (CABP) is often treated empirically. Major guidelines, such as the American Thoracic Society (ATS)/Infectious Diseases Society of America (IDSA) guideline for the management of community-acquired pneumonia recommend β-lactam, macrolide, doxycycline, or respiratory quinolone monotherapy for outpatients with nonsevere infection, whereas combination therapy is recommended for patients with comorbidities [3]. Data from the Centers for Disease Control and Prevention (CDC) Active Bacterial Core surveillance (ABCs) for *S pneumoniae* supported these recommendations [4]; however, the CDC surveillance program only includes isolates causing invasive pneumococcal disease (IPD), and large studies have demonstrated a higher burden of resistance among noninvasive isolates, specifically respiratory isolates [5]. Thus, the true burden of resistance for *S pneumoniae* may be underestimated for macrolides and tetracyclines and unknown for antibiotics not tested by the CDC including amoxicillin, doxycycline, and oral cephalosporins.

A recent systematic review and meta-analysis of randomized controlled trials reported that the clinical cure rates between the doxycycline and comparator groups (macrolides or fluroquinolones) were similar (87.2% vs 82.6%) to treat mild to moderate CABP [3, 6]. However, this meta-analysis included trials from 1984–2004, a period in which pneumococcal resistance to penicillins, macrolides, and tetracyclines was low (<3%) [7, 8]. In contrast, elevated resistance rates to penicillin (3%–14%), erythromycin (37%–45%), and tetracyclines (19%–26%) were reported in *S pneumoniae* in the United States (US) in the last 2 decades [1]. This study evaluated the in vitro activity of omadacycline and orally available comparator agents against *S pneumoniae* causing IPD and non-IPD in US hospitals during 2019–2021. Tetracycline resistance mechanisms were also evaluated in isolates with minimum inhibitory concentration (MIC) values near or above the susceptible breakpoint for doxycycline.

## MATERIALS AND METHODS

A total of 1038 *S pneumoniae* isolates recovered from adult (44.8%, 18–65 years of age; 20.3%, >65 years of age) and pediatric (32.1%, ≤17 years of age) patients in 31 medical centers in the US were included. Isolates were collected between 2019 and 2021 as part of the SENTRY Antimicrobial Surveillance Program, and tested for susceptibility by Clinical and Laboratory Standards Institute (CLSI) broth microdilution method [9] and interpreted according to current CLSI guidelines [10]. Isolates originated from patients with CABP (n = 933), bloodstream infections (n = 72), or nosocomial pneumonia (n = 33); additional information related to the SENTRY Surveillance Program and pneumonia isolates can be found as previously published [1].

A selection of 115 *S pneumoniae* isolates were subjected to molecular evaluation. These included 75 or 93.8% of isolates with doxycycline MIC results (0.25–2 mg/L) around the CLSI breakpoints (≤0.25 mg/L for susceptible, 0.5 mg/L for intermediate, and ≥1 mg/L for resistant) to correlate the current criteria with resistance mechanisms and a random collection of 40 or 21.9% of doxycycline-resistant isolates (MIC, 4 to >8 mg/L). Whole genome sequencing was performed on this subset using total genomic DNA as input material for library construction. DNA libraries were prepared using the Illumina DNA library construction protocol and index kit and sequenced on a NextSeq 1000 Sequencer (Illumina, San Diego, California) using NextSeq 1000/2000 P2 Reagents (300 cycles). Trimmed reads were error corrected using BayesHammer and de novo assembled using SPAdes version 3.15.3. An in-house designed software pipeline consisting of a database generated from the National Center for Biotechnology Information Bacterial Antimicrobial Resistance Reference Gene Database used the assembled sequences to screen for >80 acquired tetracycline resistance genes [11] and target site mutations (16S, 23S, *rpsJ*, *rpsM*, and *rplD* genes). Nucleotide and amino acid mutations were obtained by comparison with sequences from a wild-type reference control strain ATCC 49619.

Additionally, in silico serotyping was performed using PneumoCaT (Pneumococcal Capsular Typing) v.1.2.1, which uses a 2-step approach to assign capsular type to *S pneumoniae* genomic data. In the first step, if the reads matched >90% to 1 or more of the 92 serotypes for *S pneumoniae* plus 2 additional subtypes/molecular types, a capsular type was assigned. If >1 loci were matched, then in the second step, a variant-based approach that utilizes the capsular type variant database to distinguish serotypes within a serogroup/genogroup was applied to make a call on the serotype.

## RESULTS

Nearly all of the 1038 *S pneumoniae* isolates tested were susceptible to omadacycline (99.8%) when using the CABP Food and Drug Administration (FDA) breakpoint. High susceptibility rates were also observed for amoxicillin-clavulanate, ceftriaxone, and levofloxacin. Other oral agents had limited activity overall (doxycycline, 80.2%; cefpodoxime, 76.8%; oral penicillin, 63.5%; azithromycin, 54.4%) (Table 1). Among isolates screened for tetracycline resistance genes, *tet*(M)-positive isolates included all (52/52) doxycycline-resistant (MIC, ≥1 mg/L), 5 of 7 intermediate (MIC, 0.5 mg/L) and 4 of 56 susceptible (MIC, 0.25 mg/L) isolates. Additional tetracycline resistance mechanisms, such as target site mutations or acquired genes other than *tet*(M), were not identified. Only omadacycline (100% susceptible), ceftriaxone (93.6%), and levofloxacin (98.4%) were active against *tet*(M)-positive *S pneumoniae*. Cefpodoxime (68.9%) and amoxicillin-clavulanate (82.0%) had suboptimal activity against this subset. Other agents including oral penicillin (36.1%), doxycycline (6.6%), and azithromycin (3.3%) also showed very low susceptibilities against this subset. Similarly, only omadacycline (100% susceptible) and levofloxacin (99.0% to 100% susceptible) remained active against amoxicillin-clavulanic acid, azithromycin, or cefpodoxime nonsusceptible, *tet*(M) positive, and multidrug-resistant *S pneumoniae* (ie, nonsusceptibility to amoxicillin-clavulanic acid, cefpodoxime, and doxycycline) (Table 1). The susceptibility results for ceftriaxone (55.9% to 95.8% susceptible) varied across these subsets.

**Table 1.** Susceptibility Profiles of *Streptococcus pneumoniae* Isolates From US Medical Centers by Geography, Resistant Subgroups and Molecular Characteristics

Characteristic (No. Tested)	Antimicrobial Agent (% Susceptible)^a^								
	Omadacycline	Penicillin	Cefpodoxime	Tetracycline	Doxycycline	Ceftriaxone	Azithromycin	Amoxicillin-Clavulanate	Levofloxacin
Overall (1038)	99.8	63.5	76.8	79.5	80.2	98.1	54.4	95.6	99.7
US census division									
New England (130)	100	73.1	85.4	66.1	69.2	98.5	49.2	99.2	99.2
Middle Atlantic (119)	100	62.2	73.9	74.6	74.8	97.5	58.0	94.0	100
East North Central (184)	99.5	60.3	76.6	87.0	86.4	97.3	58.7	95.6	100
West North Central (114)	100	64.9	79.8	84.2	85.1	100.0	47.4	99.1	100
South Atlantic (114)	99.1	56.1	68.4	75.4	76.3	96.5	43.0	93.0	100
East South Central (71)	100	56.3	70.4	76.0	76.1	94.4	47.9	88.6	100
West South Central (150)	100	51.3	65.3	76.7	76.7	100.0	41.3	94.0	99.3
Mountain (81)	100	83.9	88.9	87.6	88.9	97.5	76.5	96.3	98.8
Pacific (75)	100	74.7	90.7	92.0	93.3	100.0	84.0	100	100
Phenotype									
Amoxicillin-clavulanate NS (45)	100	0.0	0.0	24.4	16.1	60.0	0.0	0.0	100
Azithromycin NS (473)	100	64.5	53.3	58.9	58.0	95.8	0.0	90.5	99.4
Cefpodoxime NS (241)	99.6	0.4	0.0	67.1	67.6	92.1	8.3	81.3	99.2
Doxycycline NS (205)	99.2	29.3	61.9	1.0	0.0	92.2	7.8	83.4	99.0
MDR^b^ (34)	100	0.0	0.0	0.0	0.0	55.9	0.0	0.0	100
Molecularly characterized (115)	99.1	48.7	70.4	47.8	48.7	96.6	30.4	89.6	99.1
*tet*(M) isolates (61)	100	36.1	68.9	1.6	6.6	93.6	3.3	82.0	98.4
Serotype^c^									
22F (15)	100	100	100	93.3	86.7	100	73.3	100	100
15A (14)	100	0.0	71.4	7.1	7.1	92.9	0.0	92.9	100
35B (12)	100	0.0	0.0	83.3	83.3	100	8.3	83.3	100
23A (12)	100	41.7	83.3	33.3	33.3	100	33.3	100	100

Abbreviations: MDR, multidrug resistant; NS, nonsusceptible; US, United States.

^a^Clinical and Laboratory Standards Institute (CLSI) susceptible breakpoints applied to all agents, except for omadacycline, where the US Food and Drug Administration breakpoint applied. Breakpoints applied were as follows: omadacycline, ≤0.25 mg/L; oral penicillin, ≤0.06 mg/L (nonmeningitis isolates); cefpodoxime, ≤0.5 mg/L; tetracycline, ≤1 mg/L; doxycycline, ≤0.25 mg/L; ceftriaxone, ≤1 mg/L; azithromycin, ≤0.5 mg/L; amoxicillin-clavulanate, ≤2/1 mg/L; levofloxacin, ≤2 mg/L.

^b^MDR *S pneumoniae* isolates were nonsusceptible to amoxicillin-clavulanic acid, cefpodoxime, and doxycycline per CLSI criteria.

^c^Represent most common serotypes. Other serotypes were represented by <10 isolates, as follows: serotype 3 (3 isolates), 20 (4), 38 (2), 6C (6), 7C (1), 7F (1), 9N (8), 11A (2), 12F (2), 15B/C (7), 17F (1), 19A (9), 19F (4), 23B1 (5), 28A (4), 28F (1), and nonencapsulated *S pneumoniae* group I and group II (2).

Serotyping data were also obtained for the 115 *S pneumoniae* sequenced for the screening of tetracycline resistance mechanisms, and 21 individual serotypes were noted (Table 1). Two isolates failed serotype identification, and each was categorized as nonencapsulated *S pneumoniae* group I and group II. Types 22F (13/56 [23.2%]), 35B (10/56 [17.9%]), and 6C (6/56 [10.7%]) were the most common among doxycycline-susceptible *S pneumoniae*, whereas 15A (13/59 [22.0%]), 19A (9/59 [15.2%]), 23A (8/59 [13.6%]), and 9N (5/59 [8.5%]) were common among doxycycline-nonsusceptible *S pneumoniae*.

## DISCUSSION

CABP is associated with treatment failure and is a leading infectious cause of hospitalization and death. In general, there is growing and potentially unrecognized pneumococcal antibiotic resistance to current therapies. This may be due to the lack of routine culture and isolation of *S pneumoniae*, and consequently lack of local susceptibility testing. Therefore, antimicrobial susceptibility profiles of isolates causing IPD and non-IPD are dependent on few antibiotics included in national surveillance programs. This study reports the *S pneumoniae* susceptibilities for all current antibiotic classes and formulations recommended for CABP after implementation of the 13-valent pneumococcal conjugate vaccine (PCV). Macrolides continue to exceed the 25% resistance threshold in all US census regions (41.3%–58.7% nonsusceptible to azithromycin), except the Pacific (16%) and Mountain (23.5%) regions. The overall nonsusceptibility to macrolide described here (45.6%) is higher than that reported by the CDC ABCs program (29.3%) [4], but consistent with prior reports and further supports the recommendation to not use macrolide monotherapy for CABP.

In general, doxycycline (19.8%) had a nonsusceptibility rate similar to that of tetracycline (20.5%), and an acceptable coverage (>90%) for both agents was observed only against isolates from the Pacific region (92.0%–93.3% susceptible). These results may indicate that the clinical efficacy of doxycycline may currently be compromised due to the high number of resistant isolates. The evidence for doxycycline efficacy for treating CABP remains scarce, but the most recent review by Choi et al [6] supports the doxycycline recommendation by the ATS/IDSA. However, it is important to emphasize that the review by Choi et al included older studies (1984–2004), at a time when pneumococcal resistance was negligible. The similar lack of susceptibility for doxycycline and tetracycline is likely a result of *tet*(M) being the dominant mechanism of tetracycline resistance, which conveys resistance to tetracycline, doxycycline, and minocycline. A tetracycline resistance mechanism—that is, *tet*(M)—was found in 96.6% of isolates nonsusceptible to doxycycline and in only 7.1% of those isolates having an MIC at the breakpoint for susceptibility (ie, 0.25 mg/L). These results indicate *tet*(M) as the main resistance mechanisms, aligning with prior reports [12], and that the clinical doxycycline susceptible breakpoint may be above the epidemiological cutoff and may categorize non-wild-type isolates as susceptible; however, the clinical implications for this are unknown.

Clinical guidelines recommend the selection of oral β-lactams for the treatment of CABP in outpatients with comorbidities without preference. The rationale provided is that resistance to β-lactam agents is uncommon and combination therapy is recommended for macrolide- and doxycycline-resistant *S pneumoniae*. Based upon the data herein, susceptibility rates for amoxicillin-clavulanate (95.6%) and cefpodoxime (76.8%) differ substantially, which are further reduced in the presence of a macrolide or doxycycline resistance phenotype (83.4%–90.5% for amoxicillin-clavulanate and 53.3%–61.9% for cefpodoxime). This suggests that combination regimens, particularly those with an oral cephalosporin, may provide inadequate coverage against macrolide- or doxycycline-resistant *S pneumoniae*.

Omadacycline is an FDA-approved once-daily, intravenous and oral formulation approved for adult patients with CABP [13]. In this study, omadacycline activity was not impacted by nonsusceptibility to amoxicillin-clavulanate, cefpodoxime, doxycycline, or azithromycin, which is consistent with prior analyses [13]. Finally, this study described numerous serotypes (21 among 115 isolates with decreased susceptibility to doxycycline), with 60.9% of these serotypes, including the 3 most common serotypes noted here (15A, 35B, and 23A), not being covered by 15-valent PCV (PCV15) or 20-valent PCV (PCV20), or 48.6% not being covered by any PCV or pneumococcal polysaccharide vaccine. In addition, the most common serotype, 22F, was previously associated with high health and economic burden [14], although now covered by both PCV15 and PCV20 vaccines. Similar to other phenotypic and genotypic characterized isolates, omadacycline was active against strains belonging to high-burden serotypes. It is important to mention that this study included a broad collection of contemporary isolates from the US, and the data presented here can be useful when evaluating options for treating infections caused by *S pneumoniae* with various phenotypes and genotypes. However, a small subset of isolates was selected for molecular characterization, and the selection of these isolates was based on the doxycycline MIC results.

## CONCLUSIONS

The oral antimicrobial options recommended for CABP showed low susceptibility rates against *S pneumoniae* from the US, except against isolates from the Pacific region. *Tet*(M) remains the dominant tetracycline resistance mechanism, against which omadacycline retained potent activity. These contemporary data provide helpful national and regional guidance for the empiric oral treatment options for CABP. In addition, these data support omadacycline as an important empiric treatment option including against isolates that are nonsusceptible to other drug classes including tetracycline and doxycycline.

## Notes

***Author contributions.*** L. M. D.: data curation, formal analysis, writing–original draft. M. D. H.: conceptualization. S. C.: investigation. M. C.: project administration. R. E. M.: conceptualization, project administration, writing–review and editing.

***Data availability.*** Data are available upon reasonable request.

***Patient consent.*** Patient consent was not applicable to monitoring surveillance studies when obtaining data from frozen, stored organisms and due to the retrospective nature of the study.

***Financial support.*** This study has been funded in part by federal funds from the Biomedical Advanced Research and Development Authority, US Department of Health and Human Services (contract number 75A50120C00001) and by Paratek Pharmaceuticals, Inc. This study was performed by JMI Laboratories and supported by Paratek Pharmaceuticals, which included funding for services related to preparing this manuscript.
